# Progress in the Understanding and Applications of the Intrinsic Reactivity of Graphene‐Based Materials

**DOI:** 10.1002/smsc.202000026

**Published:** 2020-12-18

**Authors:** Zahra Komeily-Nia, Liang-Ti Qu, Jing-Liang Li

**Affiliations:** ^1^ Institute for Frontier Materials Deakin University Geelong Victoria 3217 Australia; ^2^ Department of Chemistry Tsinghua University Beijing 100081 P. R. China

**Keywords:** biosensing, catalysis, free radicals, graphene, graphene oxide, nanocomposites, oxygen reduction reaction

## Abstract

Enhancing the intrinsic reactivity of graphene materials is essential for the development of low‐cost materials such as catalysts for various applications. Although an increasing understanding of the intrinsic reactivity of these materials is being achieved, the mechanisms of these materials for catalyzing various reactions have not been fully understood. It is believed that the intrinsic reactivity of pristine graphene originates from its edge and defect sites, and unpaired electrons (radicals) particularly of graphene oxide have also been demonstrated to contribute to the reactivity. Herein, the various edges and defects, and radicals, as well as their influences on the electron structure, reactivity, and applications of graphene‐based materials are reviewed and analyzed. Knowledge gaps in advancing the understanding of the structure–property–reactivity correlations of these materials are discussed.

## Graphical abstract

The intrinsic reactivity of graphene materials can be enhanced by engineering its edge, defects, and free radicals, which enables fabrication of low‐cost carbon‐based metal‐free and dopant‐free catalysts for various applications.

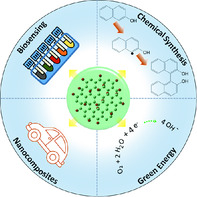

## 1 Introduction

Graphene‐based materials, including pristine graphene, graphene oxide (GO), and reduced graphene oxide (rGO), are useful for various applications such as electronic devices and energy storage,^[^ ^1^ ^]^ clean water production,^[^ ^2^ ^]^ environmental remediation (e.g., adsorption of oil spill),^[^ ^3^ ^]^ photo‐ and electrocatalysis for green energy conversion,^[^ ^4^ ^]^ composites reinforcement,^[^ ^5^ ^]^ antimicrobial uses,^[^ ^6^ ^]^ and nanotheranostics.^[^ ^7^ ^]^ Some of the applications take advantage of the apparent physical (e.g., conductivity, hydrophobicity, and surface area) and chemical (e.g., functional groups) properties of the materials, while others rely on their reactivity that is dependent not only on their chemical but also on electronic properties (e.g., catalytic applications).

Pristine graphene with a perfect honeycomb structure is an inert material. However, in reality, graphene synthesized with different physical and chemical methods contains various defects. The defects include topological defects (edges and holes) and vacancy defects (nonhexagonal rings), which are reported to have electronic structure different from the basal plane of graphite/graphene. The dangling groups and valences at the defects affect the charge distribution and often lead to the creation of localized unpaired electrons (radicals).^[^ ^8^ ^]^ However, for pristine graphite/graphene, the intrinsic activity due to limited defects is far below the traditional metal‐based catalysts and therefore not practically useful. To produce reactive graphene materials, doping with electron‐withdrawing elements such as nitrogen, boron, phosphorous, or a combination of them, which alter their electron structures, has been generally adopted.^[^ ^4^, ^9^ ^]^ The efficiency of doped graphene was found comparable or even superior to those of Pt‐based catalysts for oxygen reduction reaction (ORR) in fuel cells.^[^ ^10^ ^]^ The mechanism of enhancement is that the doped atoms can modify the charge distribution of neighboring carbon atoms to facilitate oxygen adsorption and charge transfer to the oxygen adsorbed.^[^ ^11^ ^]^ However, doping with atoms usually requires harsh processing conditions such as a high temperature (e.g., 300–800 °C).^[4b]^ Moreover, the level of doping has to be precisely controlled to get the optimal efficiency because it has been observed that a higher nitrogen doping does not always give a higher efficiency.^[^ ^12^ ^]^ Therefore, growing interest is being shown on the enhancement of the intrinsic reactivity of graphene materials by controlling their electronic properties without element doping. A growing number of studies on deliberate control of the intrinsic reactivity of graphene‐based materials by increasing their edges and defects, rather than doping, have been reported in the last few years.^[^ ^12^, ^13^ ^]^ Increasing evidence from a few recent studies demonstrated that proper defect engineering can confer graphene‐based catalysts with catalytic performance similar to or even superior to doped ones.^[^ ^14^ ^]^ Defect engineering is a promising approach to producing dopant‐ and metal‐free carbon‐based catalysts. It is believed that the reactivity of the edges and defects is due to the localization of electrons and frequently unpaired electrons (radicals). However, the radical properties of the materials were mostly not characterized and integrated into the understanding of the mechanisms of the different reactions promoted by these materials.

Apart from defects engineering of graphene, GO, a derivative of graphene with significantly high contents of defects and edges, may also offer opportunities for achieving high efficiency catalysts without chemical doping. Strictly, GO is also doped with oxygen, but as an intermediate for graphene production, it is generally regarded differently from the conventional element doping where the heteroatoms are introduced purposely through thermal or plasma treatment of graphene. The intricate/heterogeneous chemistry and structure, and therefore the electronic properties of GO (and rGO), which depend on the synthetic and posttreatment conditions, contribute to the inconsistent or even contradictory observations regarding their effectiveness for some applications such as antimicrobial uses. Despite the significant and increasing efforts devoted to the studies of GO, more investigations are needed to understand the correlations between their physical/chemical structure and electron structure, as well as how the structures affect their reactivity.

It is worth mentioning that apart from heteroatom doping, defect engineering, and oxygenation, some other approaches have been proposed for the activation of graphene. For example, it has been reported that applying a moderate mechanical strain of graphene could enhance its chemical reactivity to stabilize gold clusters, which reversed the charge transfer between the gold clusters and graphene, reducing the reaction barrier of CO catalytic oxidation from around 3.0 eV (without strain) to less than 0.2 eV (with a strain of 5%).^[^ ^15^ ^]^ A substrate engineering approach, which introduces a defect, either a substitutional impurity atom (e.g., Au, Cu, Ag, Zn) or a single vacancy, in the underlying Ru (0001) substrate that supports graphene, has also been used to improve the reactivity of graphene for CO reduction.^[^ ^16^ ^]^ It is of note that both studies are based on first‐principles calculations and experimental studies are needed to support the conclusions. A recent review for theoretical understanding of various types of graphene‐based catalysis is available.^[^ ^17^ ^]^

Although there are different ways to activate graphene materials, this current review focuses on the progress of the understanding and engineering of the intrinsic reactivity, due to edge, defects, and radicals, of graphene‐based materials for different applications. The intrinsic activity of graphene edge and defects, and radical‐carrying GO and their implications for applications including electrocatalytic energy conversion reactions, chemical bond formation, polymerization, catalytic biosensing, as well as catalytic degradation of environmental pollutants and antimicrobial applications are covered. Prior to the review of the applications, the different edges and defects, and their influences on the electron structure of graphene, the chemical structure of GO and its correlation to its radical property will be reviewed on the basis of both experimental and theoretical investigations. To the best of our knowledge, this is the first article that reviews the intrinsic reactivity (nondoping) of graphene materials and its applications.

## 2 Types of Edge and Defects, and Their Influence on the Electron Structure of Graphene

The extended *π* conjugated electron structure of carbon materials has been of interest to scientists, particularly after the advent of novel carbon materials including fullerene, carbon nanotubes (CNTs), and graphene. Graphite and graphene have been regarded as inert materials. In the inner plane of a pristine graphene, each carbon is covalently bonded through three electrons to three neighboring carbon atoms, and the fourth valence electrons (*p*_*z*_) are delocalized to form half‐filled *π* orbitals that permit free movement of electrons. Therefore, the inner plane of graphene is charge‐neutral. However, in reality, graphene materials have many defects such as the boundary edge sites, holes, and vacancy‐like defects, which are created during their preparation. Compared with the carbon in the inner plane, a carbon atom on the edge has only two neighboring carbon atoms. Therefore, the electron distribution at the edge is different from that in the inner plane, and the free electrons at the edge are often localized and unpaired, making the edge chemically reactive.[^8^, ^18^] The other types of defect also disturb the electron distribution. Raman spectroscopy is a powerful tool to characterize the degree, size, and nature of defects.^[^ ^19^ ^]^

**Figure 1:**
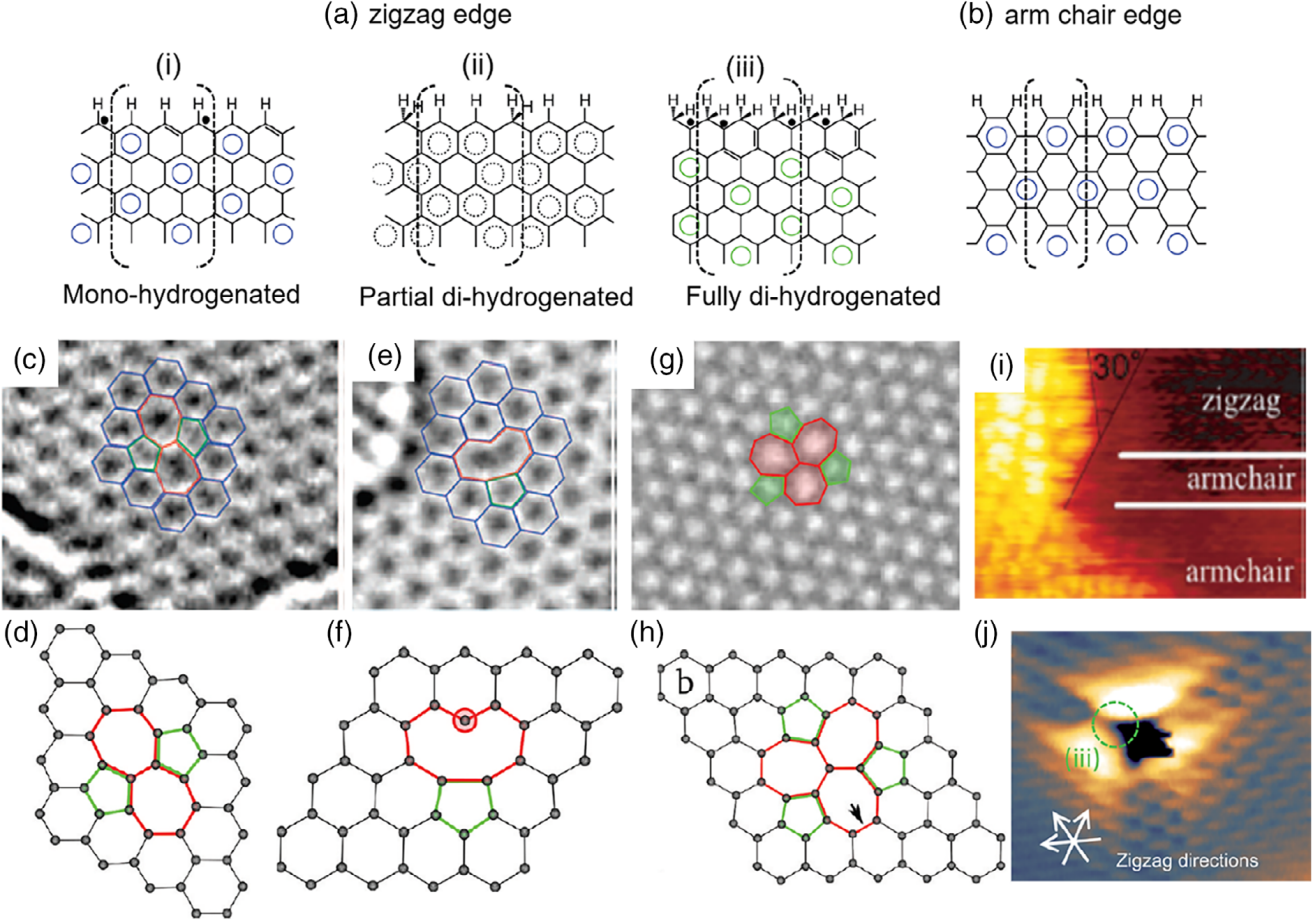
a) Hydrogenated zigzag edges and b) armchair edge black dots indicate radicals. c,d) SW defect, e,f) single vacancy defect, and g,h) double vacancy (555–777) defect. STM images of i) edges and j) a hole on graphene. (a,b) Reproduced with permission.^[21a]^ Copyright 2014, The Royal Society of Chemistry; (c,e) Reproduced with permission.^[^ ^105^ ^]^ Copyright 2008, The American Chemical Society; (d,f,g,h) Reproduced with permission.^[^ ^106^ ^]^ Copyright 2011, The American Chemical Society; (i) Reproduced with permission.^[^ ^107^ ^]^ Copyright 2005, The American Physical Society; (j) Reproduced with permission.^[21a]^ Copyright 2014, The Royal Society of Chemistry.

Electron paramagnetic resonance (EPR), also called electron spin resonance (ESR) spectroscopy, is a convenient technique to characterize the number of unpaired electrons, not only at the edge but also at the valence defects. Pristine graphene/graphite has a very weak and broad resonance signal (broad Dysonian line shape) with a *g* factor of 2.007–2.008, attributable to the intrinsic delocalized (conducting) *π* electrons in the graphite sheets. Increasing the edges of graphite through ball‐milling can lead to a significant increase and narrowing of the spin density with *g* factor at 2.003, typical of carbon radicals.^[^ ^20^ ^]^ This indicates that increasing the boundary edges of graphite could enhance its reactivity. Furthermore, the edge reactivity depends on the geometrical shape and chemistry of the edge. When graphene is cut along its crystallographic directions and hydrogen atoms are attached to each edge carbon atom, two types of edges, which are zigzag and armchair edges, can form (**Figure** **1**a,b). The zigzag edge of graphene supports the localized *π* state (edge state) with chemically reactive radical characters.^[^ ^21^ ^]^ In contrast, the armchair edge does not possess the edge state and energetically stable. The electronic properties of graphene edges are also significantly affected by the binding of foreign chemical species such as hydrogen; for example, the edge localized state may disappear when every third zigzag edge site is dihydrogenated while the other two sites remain monohydrogenated, while a fully dihydrogenated edge has more radicals (Figure 1a). Compared with its basal plane, the edges, in particular, the zigzag edges of graphene, have been reported to be more reactive,^[^ ^13^, ^22^ ^]^ and edge‐rich graphene has been demonstrated to be highly efficient metal‐free electrocatalysts.^[^ ^13^ ^]^

Apart from boundary edges, holes, Stone–Wales (SW) defect, and vacancy defects are also intrinsic defects that can be created during the preparation of graphene. The holes are bounded by inner edges which are either zigzag or armchair configurations. A SW defect, created by a single pair of carbon atoms, leads to the formation of adjacent pairs of pentagonal and heptagonal rings (Figure 1c,d). The formation of a SW defect does not result in any introduction or removal of carbon atoms or dangling bonds. A single vacancy defect is created when one carbon atom misses from a hexagonal ring, which results in a dangling bond (Figure 1e,f). However, a double vacancy defect forms when two carbon atoms are kicked off a ring, leading to the formation of multiple nonhexagonal rings (Figure 1g,h). SW, single vacancy, and double (or multiple) vacancy defects are considered as point defects. Line defects with strings of defects are frequently formed during chemical vapor deposition (CVD) growth of graphene on a substrate, due to crystallographic mismatch between graphene pieces growing from adjacent domains.^[^ ^23^ ^]^ This has been the big challenge for creating graphene of big size that is single crystalline.

Local probing techniques such as scanning transmission electron microscopy (STEM) and scanning tunneling microscopy (STM) are powerful tools to characterize topography, defects, and density of charge carriers at the atomic level. The zigzag and armchair structure, as well as defects, can be clearly imaged with these techniques. The localization of electrons on the zigzag edges and defects of graphene can increase the tunneling current, which leads to bright spots on a STM image (Figure 1i,j).

The influences of edges and defects on the electronic properties of graphene have been studied theoretically and experimentally.[^8^, ^24^] Heteroatom doping which replaces carbon atoms of graphene with elements such as nitrogen, sulfur and boron also creates defects, which has received extensive investigations.[^4^, ^9^, ^25^] As this work focuses on the reactivity arising from the intrinsic defects, the influence of doping will not be covered, except in some cases where a combination of doping and defect engineering was used.

## 3 Edge/Defect Reactivity of Graphene Materials and Applications

Graphene materials are mostly used as cocatalysts of the conventional metal‐based catalysts, due to their excellent ability to accept and transport photogenerated electrons,^[^ ^26^ ^]^ or doped with heteroatoms.^[^ ^9^, ^27^ ^]^ Enhancing their intrinsic activity may make it feasible to eliminate the doping process. Tuning the electron properties of these materials promises the design of efficient graphene‐based metal‐free catalysts.

Due to the important use of graphite materials for electrocatalytic electrodes, the electron transfer kinetics at the edge of graphite has been characterized as early as late 1980s.^[^ ^28^ ^]^ It was observed that increasing the graphitic edge plane of highly ordered pyrolytic graphite (HOPG) was necessary to achieve faster electron transfer (e.g., six orders of magnitude increase for dopamine redox system).^[28a]^ The last decade showed renewed interest in the electrochemistry of graphite/graphene edge and defects due to the rapid growth in the applications of these materials. A number of works in the last few years have demonstrated that the edge and defects of graphene‐based materials are effective catalytic sites,^[^ ^12^, ^14^, ^29^ ^]^ the activity of which is even higher than nitrogen‐doped graphene and metallic catalysts. Interest in the influences of edge/defect on the activity of graphene materials has been mainly in the area of electrocatalytic energy conversion reactions such as ORR, oxygen evolution reaction (OER), and hydrogen evolution reaction (HER), which are important reactions for green energy conversion (e.g., water splitting, metal–air battery, and fuel cells),^[^ ^30^ ^]^ though they have also been found to benefit other applications such as energy storage (e.g., supercapacitors[^24^, ^31^] and hydrogen storage^[^ ^32^ ^]^) and dye‐sensitized solar cell,^[^ ^33^ ^]^ attributable to the enhanced electron transfer by the defects. Therefore, this section focuses on the findings of edge/defect activity in these catalytic energy conversion reactions.

### 3.1 Edge Effects

**Figure 2:**
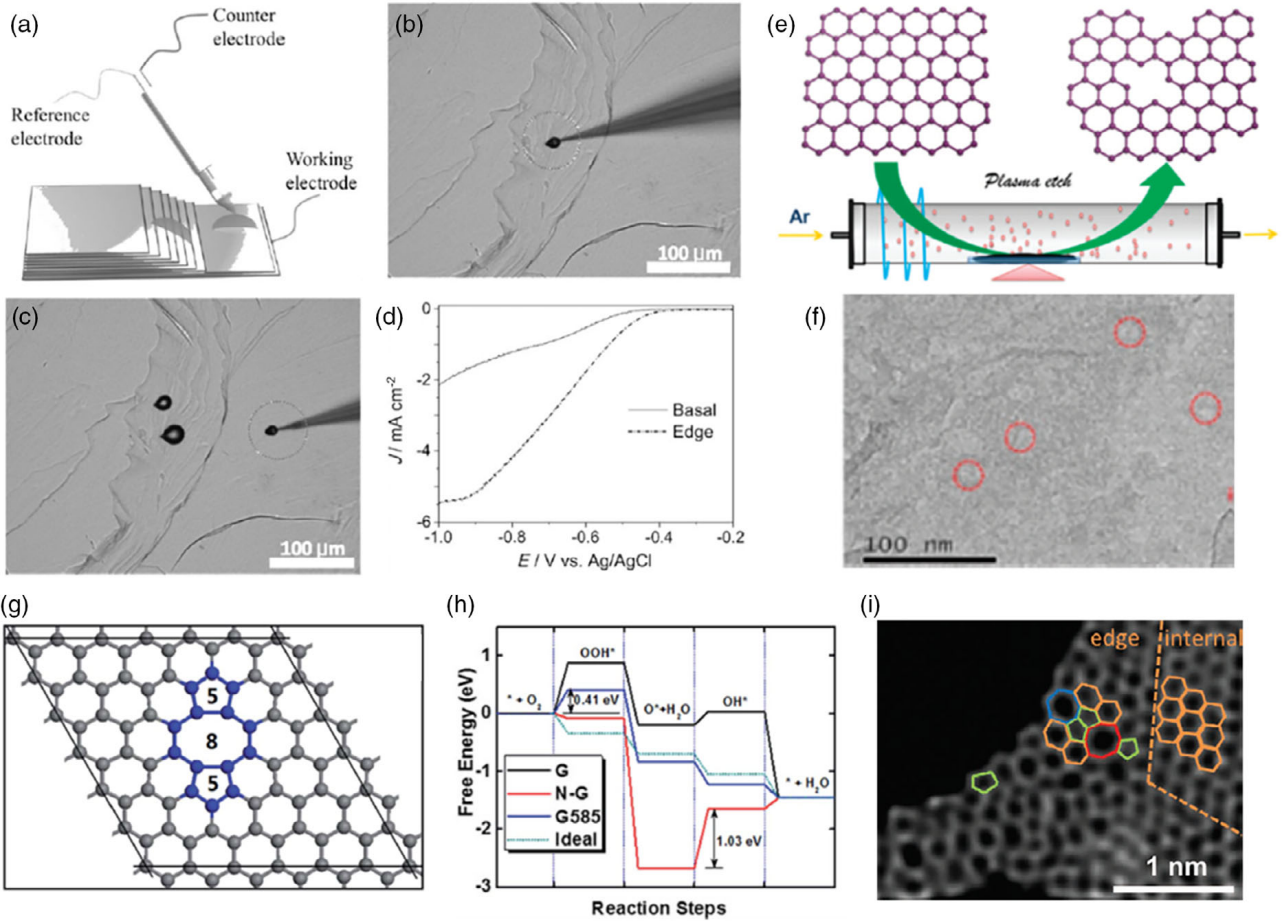
a) Microapparatus for the ORR electrochemical experiment on air‐saturated droplet deposited on b) edge of HOPG and c) basal plane of HOPG. d) LSV curves of the ORR tested for a droplet located either on the edge of the HOPG. e) Schematic description of Ar plasma etching to prepare edge‐rich graphene. f) High‐resolution TEM of plasma‐treated graphene. g) Schematic of G585 vacancy defect. h) Calculated free energy diagram of perfect monolayer graphene (G), N‐doped graphene (N‐G), graphene with G585 defects (G585), and an ideal catalyst (Ideal) for the ORR at the equilibrium potentials. i) STEM image of defect graphene with hexagon, pentagon, heptagon, and octagons rings. (a–d) Reproduced with permission.^[^ ^22^ ^]^ Copyright 2014, Wiley‐VCH GmbH; (e,f) Reproduced with permission.^[^ ^13^ ^]^ Copyright 2016, The Royal Society of Chemistry; (g,h) Reproduced with permission.^[14b]^ Copyright 2015, The Royal Society of Chemistry; (i) Reproduced with permission.^[^ ^12^ ^]^ Copyright 2016, Wiley‐VCH GmbH.

Direct evidence that the edge is more active than the basal plane was obtained by conducting ORR in a droplet (**Figure** **2**a), which was deposited either on the edge (Figure 2b) or on the basal plane (Figure 2c) of HOPG.^[^ ^22^ ^]^ Linear sweep voltammetry (LSV) demonstrated that ORR on the edge proceeded with a more positive onset potential and a higher current density than that on the basal plane (Figure 2d), indicating that the edge of graphite is more active. To further demonstrate the edge effects, graphite and CNTs were ball‐milled to create more exposed edges. Those edge‐rich graphite and CNTs also demonstrated significantly enhanced ORR activity. Density functional theory (DFT) calculations verified that charged edge carbon atoms contributed to the higher ORR activity. In a more recent study, ball‐milled graphite was proved to have a significantly higher amount of unpaired electrons, mainly on the zigzag edge sites, and the electron charge transfer from the edge‐rich carbon electrode to the oxygen molecule occurs at a lower potential (0.13 eV).^[24e]^

Edge‐rich and dopant‐free graphene has been fabricated with Ar plasma etching (Figure 2e). A great number of internal holes (hole and boundary edges) on graphene were created after the plasma treatment (Figure 2f).^[^ ^13^ ^]^ The edge‐rich graphene showed excellent ORR activity. LSV characterizations demonstrated that the onset potential and half‐wave potential of the edge‐rich graphene shifted (compared with untreated graphene) positively from 0.806 and 0.572 to 0.912 and 0.737 V (electrolyte O_2_‐saturated 0.1 m KOH), respectively. The electron transfer number of edge‐rich graphene obtained from the slopes of Koutecky–Levich plots at 0.4 V was 3.85, compared with 2.31 of the untreated graphene. The higher electron transfer number of the ORR on edge‐rich than that on untreated graphene indicates more efficient reaction kinetics (one‐step and four‐electron pathway) of the ORR on the edge‐rich graphene. DFT calculations also demonstrated that the basal plane carbons carry negligible charge, while higher charge densities were observed on the edge carbons. The edge‐rich graphene showed a larger oxygen absorption energy. Based on quantum mechanics, the mechanism of ORR on dopant‐free graphene was investigated.^[^ ^34^ ^]^ It was found that ORR starts with OO chemisorbing onto the carbon edges, rather than the basal plane face, which is not energetically favorable. The binding energies of the zigzag edge tend to be stronger than those of the armchair edge, and are usually higher than those for Pt and Pt3Ni, while those for the armchair edge are lower.

Graphene nanoribbons with zigzag edges were prepared by cutting multiwall CNTs for electrocatalysis of ORR in proton exchange membrane fuel cells.^[^ ^35^ ^]^ A peak areal power density of 0.161 W cm^−2^ and a peak mass power density of 520 W g^−1^, superior to most nonprecious metal electrocatalysts, were achieved by the zigzag carbon catalyst. It also demonstrated improved stability in comparison with a representative iron–nitrogen–carbon catalyst. DFT calculation coupled with experimentation reveals that the zigzag carbon atom is the most active site for ORR among several types of carbon defects on graphene nanoribbons in an acid electrolyte.

3D graphene networks with a high density of sharp edges were synthesized with a one‐step CVD method for HER.^[^ ^36^ ^]^ The 3D graphene networks offered an extremely low onset potential of about 18 mV in a 0.5 m H_2_SO_4_ solution, and demonstrated good stability. A combination of control experiments and DFT investigations indicated that the exceptional H_2_ evolution performance was attributable to the abundant sharp edge sites of the network, which promoted the adsorption and reduction of protons. The HER performance of the material is comparable to those of transition‐metal‐hybrid or heteroatom‐doped carbon electrocatalysts.

A strategy that combines atom doping and defects creation has also been used to improve the electrocatalytic efficiency of graphene materials. For example, nitrogen‐doped, oxygen functionalized, defect and edge‐rich graphene was formed vertically on carbon cloth through in situ microwave plasma‐enhanced CVD for OER.^[^ ^37^ ^]^ The material drastically enhanced OER activity with an overpotential of 351 mV and a Tafel slope of 38 mV dec^−1^ in alkaline solution, which is even superior to the state‐of‐the‐art RuO_2_ electrocatalysts. The high performance was contributed to the several features of the material, including well‐dispersed graphene sheet without aggregation to give a large surface area and efficient gas contact with the electrolyte and efficient gas release, as well as the electron structure engineering with nitrogen‐doping, oxygen functionalization, and edges/defects. Effort was not made to isolate the contributions of the various factors.

### 3.2 Vacancy Defects

Apart from edge effects, creating vacancy defects has also been demonstrated effective for promoting catalytic reactions. First‐principles DFT calculations indicate that a type of 585 defect on graphene (G585) (Figure 2g) is more effective than N‐doping for the ORR.^[14b]^ Although nitrogen‐doped graphene (N‐G) can promote the adsorption of oxygen molecules (O_2_ → OOH*), however, a high energy input (1.3 V) is required to reduce adsorbed oxygen molecules (O_2_ → O*) (Figure 2h), which creates a rate‐limiting step. G585 defects can also assist the adsorption of oxygen molecules, and all of the following reactions in ORR over G585 are thermodynamically favorable, being close to the ideal catalyst. To verify the DFT calculations, nitrogen‐enriched carbon was thermally treated at different temperatures (700–1000 °C) to partially remove nitrogen to create carbon with vacancy defects, which were supposed to be G585 due to the high stability of this type of defect. It was observed that carbon with a lower N content (more defects) exhibited better ORR performance, which means a higher nitrogen doping may not give a higher ORR catalytic efficiency, and instead, the presence of suitable defects has more significant influence. Both the onset potential and current density were significantly improved with the removal of N, and C‐1000 (treated at 1000 °C) with 0.56 at% N shows the highest activity. Interestingly, when oxygen was introduced to the C‐1000 sample to further remove nitrogen (0.21 at%), the sample (C‐1000‐O_2_), with a significant amount of defects, displayed superior catalytic reactivity, almost comparable to the commercial Pt/C catalyst. In a following study from the same research group, graphene with various defects (e.g., pentagons, heptagons, and octagons) was created also by removing nitrogen atoms from nitrogen‐doped graphene. The creation of these defects was demonstrated by high‐resolution images in this work (Figure 2i). The catalysts demonstrated to be trifunctional for ORR, HER, and OER.^[^ ^12^ ^]^ The defective graphene was used as a catalyst for Zn–air battery, displaying very stable charge and discharge voltages, high current, and power density, which is comparable to Pt. Both studies indicate that creating defined vacancy defects is crucial for producing efficient graphene‐based catalysts. Synergistic effects of a hybrid catalyst coupling exfoliated Ni–Fe layered double hydroxide (LDH) nanosheet (NS) and defective graphene (DG) for OER and HER were also reported by Jia et al.^[^ ^38^ ^]^ For example, the overpotential (0.21 V) of catalytic OER is among the lowest of non‐noble metal catalysts and the Tafel slope is 52 mV dec^−1^, which is much lower than that of the Ir/C catalyst (109 mV dec^−1^). The remarkable OER and HER performance of the hybrid catalyst was ascribed to the defective sites (DG‐5, DG‐585, or DG‐7557) on DG, which provide more efficient anchor sites to directly and strongly couple transition metal atoms (Ni and Fe) on 2D exfoliated NiFe LDH nanolayers, leading to significantly enhanced density of electron transfer at specific domains.

While most of studies have focused on the mechanism of heteroatom doping for catalytic reactions, the contributions of defects in the materials have not been well understood. Heteroatoms such as nitrogen are electron‐withdrawing. Adding such an atom is considered to change the charge of the neighboring carbon atoms. The removal of a carbon atom from the graphene structure may have a bigger impact on its electron structure than adding a heteroatom.^[14b]^ The role of intrinsic defects for electrocatalytic carbon dioxide reduction reaction (ECRR) was evaluated using nitrogen‐doped carbon catalysts (carbon spheres) with various degrees of defects.^[24a]^ A positive correlation was observed between the ECRR performance of the carbon catalysts and the content of intrinsic carbon defects. Furthermore, it was demonstrated that defective porous carbon catalysts without heteroatom dopants also had excellent catalytic performance for ECRR. The results also indicated that heteroatom doping perhaps is less important than previously envisaged for achieving a high ECRR performance.

### 3.3 Holey Graphene/Graphene Nanomesh

Graphene with a considerable number of nanosized holes is called holy graphene or graphene nanomesh. The holes provide internal edges that increase the activity of graphene materials, similar to the boundary edges. It is listed as an individual section in this review because the holes can provide additional benefits such as increase in surface area and facilitation of mass transport which are also important for catalytic applications.

**Figure 3:**
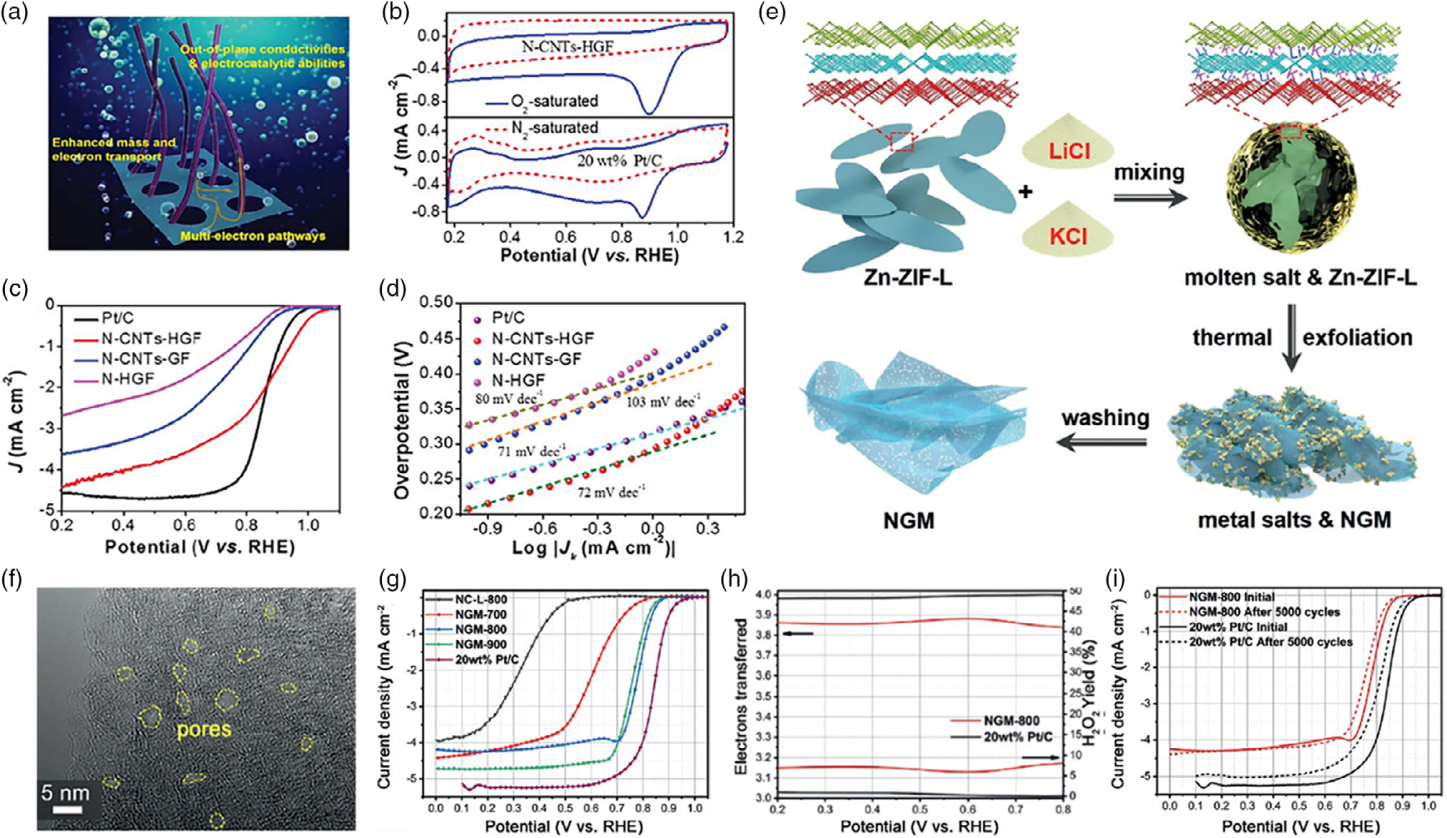
a–d) Schematic structure and mechanism of the CNT‐holey graphene. e–i) Defect‐rich graphene nanomesh catalyst obtained by thermal exfoliation of MOFs for enhancing ORR. (a–d) Reproduced with permission.^[39]^ Copyright 2016, The Royal Society of Chemistry; (e–i) Reproduced with permission.^[^ ^40^ ^]^ Copyright 2019, Wiley‐VCH GmbH.

Qu et al. developed a highly efficient ORR catalyst by in situ, precisely positioning the growth of nitrogen‐doped CNTs on the holey edges of porous graphene sheets (N‐CNTs–HGF) (**Figure** **3**).^[^ ^39^ ^]^ The interconnected pore‐rich framework not only increased the active sites in the nanopores, but also improved mass transfer in the macropores. In addition, the structure not only enhanced the electronic conductivity because of the synergistic effect, which forms efficient multicharge transfer pathways, but also unprecedentedly shortened the distance of the mass transfer and promoted the rate of charge transport along the interfaces of holes on graphene and CNTs (Figure 3a). This well‐controlled structure N‐CNTs–HGF exhibits a pronounced oxygen reduction peak at ≈0.90 V in an oxygen‐saturated KOH solution, which surpasses the state‐of‐the‐art commercial Pt/C catalyst of ≈0.873 V (Figure 3b). LSV for the N‐CNTs–HGF catalyst revealed a positive onset potential of ≈1.08 V, which is also better than Pt/C (1.05 V), and far higher than that of N‐CNTs–GF (without holes) (0.97 V) (Figure 3c). The N‐CNTs–HGF catalyst exhibits a Tafel slope of ≈72 mV dec^−1^, which is close to that of Pt/C (71 mV dec^−1^), and much lower than N‐CNTs–GF (103 mV dec^−1^), thus indicating faster charge and mass transport. The potential of the catalysts as an efficient air cathode in Zn–air batteries was also demonstrated.

Defect‐rich graphene nanomesh (holey graphene) was produced by thermal exfoliation of metal–organic frameworks (MOFs) for ORR.^[^ ^40^ ^]^ Very thin (1.3 nm) graphene mesh was obtained by a two‐step dimensional reduction of Zn‐containing zeolite imidazolate framework nanoleaves (Zn‐ZIF‐L) (Figure 3e). The material has a unique ultrathin 2D morphology, high porosity, rich and accessible nitrogen‐doped active sites, and defective graphene edges (Figure 3f). The nanomesh was doped with nitrogen. Thermal treatments (700, 800, and 900 °C) were used to tune the nitrogen contents and defects. The nitrogen contents of these samples are 11.54, 4.68, and 2.82 at%. It was observed that the nanomesh treated at 800 °C gave the best performance. It displayed an unprecedented ORR activity with an impressive onset potential of 0.860 V, half‐wave potential of 0.781 V, and current density of 4.243 mA cm^−2^ (Figure 3g), which are close to those of commercial Pt/C electrode. The ORR follows a four‐electron pathway in an acidic electrolyte (Figure 3h). The high catalytic activity of the nanomesh was attributed to its ultrathin 2D morphology with hierarchical pores as well as defect‐enriched structure. The unique low dimensional and porous structure ensures fast mass transport and sustainable exposure of active sites, and prevents the deactivation of active sites that would be otherwise induced by the restacking of carbon nanosheets or the blocking of transfer pathways. The nanomesh also demonstrated high recyclability (Figure 3i), without an obvious change in performance after 5000 cycles of use. The contribution of nitrogen‐doping to the overall performance of the nanomesh was not evaluated. However, the results did indicate that a higher nitrogen doping may not benefit the catalytic activity.

## 4 Methods to Create and Control Edges/Defects

As discussed earlier, to enhance the intrinsic reactivity of graphene material, it is important to create more edges, in particular zigzag edges, and defects that have demonstrated high reactivity. Compared with topological defects, the creation of vacancy defects has not been well reported. As discussed in Section 3.2, doping with heteroatoms (to replace carbon atoms) followed by removal of the atoms is a promising way to create some vacancy defects.^[^ ^12^, ^14^ ^]^ The different preparation methods such as CVD and chemical oxidation/reduction can create different defects on graphene. However, controlled formation of defined vacancy defects is more difficult than the topological defects, which needs more investigations. Here, we will discuss the different methods used to create topological defects (edges and holes). The methods can be classified into bulk and localized methods, according to the amount of samples they can treat and the resolutions of treatment.

### 4.1 Bulk Creation of Topological Defects

These methods are suitable to mass production of samples, but control of edge geometry is a challenge. The simplest method to increase edges is to break the materials into small pieces or make them holey. Mechanical granulation of graphite using ball‐milling is a facile method to break graphite, which does increase the activity of the materials.^[20b]^ The limitation is it is hard to get very thin graphite or graphene.

Oxidation has been generally used to exfoliate graphite to get GO, which is subsequently reduced to get graphene. A higher degree of oxidation can lead to smaller graphene materials, with increased edges. However, as to be discussed later, the reactivity of GO and rGO strongly depends on their chemistry. Smaller GO with a large fraction of edges may not be more active. A combination with chemistry control is needed. Plasma etching using Ar has been demonstrated to be effective to increase the edges of graphene, CNTs, and few‐layer graphene.^[^ ^13^ ^]^ Significant increase in edges of the basal plane was achieved by appropriate control of temperature and treatment time. The most convenient method to produce graphene mesh is to manipulate the pores of rGO in its basal planes, through chemical and thermal treatments.^[^ ^41^ ^]^ Carbonization of MOFs has also been used to prepare graphene meshes that are doped with functional heteroatoms and/or metals (can be removed by posttreatments). One challenge of this approach is to produce very thin meshes.

**Figure 4:**
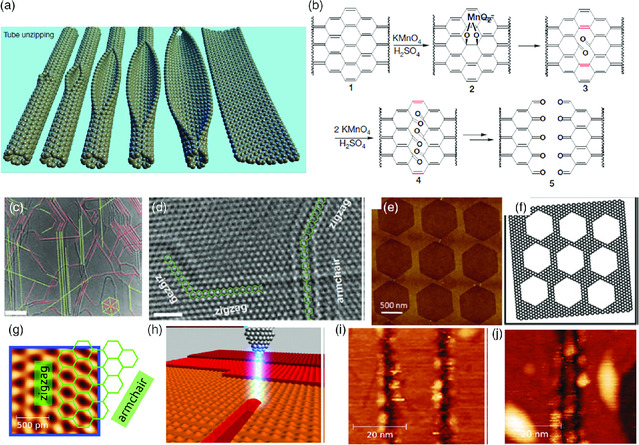
a,b) Ar plasma cutting of CNTs to get graphene nanoribbon. Reproduced with permission.^[42b]^ Copyright 2009, Springer Nature. c,d) Reconstruction of edges with a TEM–STM system. Reproduced with permission.^[^ ^45^ ^]^ Copyright 2009, The American Association for the Advancement of Science. e,f) Graphene honeycomb network formed by a combination of E‐beam and hydrogen plasma etching. Reproduced with permission.^[^ ^46^ ^]^ Copyright 2011, Wiley‐VCH GmbH. g–j) STM lithography to achieve graphene nanoribbons with desirable edge structures and widths. Reproduced with permission.^[^ ^48^ ^]^ Copyright 2014, Elsevier.

Unzipping CNTs has been successful for creating edge‐rich graphene nanoribbons^[^ ^35^ ^]^ by plasma (Ar) etching or chemical cutting.^[^ ^42^ ^]^ Illustration of the chemical unzipping is shown in **Figure** **4**a,b. Interestingly, the unzipping occurs preferably along the longitudinal direction of CNTs, which leads to the formation of well‐controlled smooth edges. Calculations based on ab initio DFT indicates that for the oxidative treatment, the unzipping starts with the potassium permanganate attacking one of the internal C—C bonds of the CNTs, stretching and breaking it. The created defect weakens neighboring bonds along the length of CNTs, making them energetically prone to be attacked too, and the results suggested that zigzag edges form during chemical unzipping of CNTs.^[^ ^43^ ^]^ Formation of zigzag edges was also suggested in experimental investigations for both plasma and chemical unzipping,^[^ ^35^, ^42^ ^]^ although direct evidence was not provided by high‐resolution images, which has been attributed to the high roughness of the edges.

### 4.2 Localized Creation of Topological Defects

Lithography by electron beam (e‐beam), plasma, and STM enables high‐resolution control of nanostructures. They have been used for reconstruction of the edges of graphene, or fabrication of graphene nanostructures with defined edges. These localized treatments that lead to well‐controlled zigzag edges are very useful for the fabrication of graphene‐based devices. The zigzag edge offers interesting properties to graphene materials, for example, graphene nanoribbons with zigzag edges demonstrated quasi‐metallic electrical transport independent of the ribbon width, a reduced edge scattering effect for charge carriers, and a characteristic phonon mode.^[^ ^44^ ^]^ Some important work is summarized later. Although they might not be very useful, due to the poor scalability, for the (chemical, electrochemical, biological) reactivity‐based applications discussed in this review, they are important to fabrication of nanoelectronic devices for applications such as electrochemical immunoassay and biosensing.

An integrated TEM–STM system was used for controlled formation of smooth edges that are with either zigzag or armchair configuration at the atomic scale with Joule heating.^[^ ^45^ ^]^ An individual nanoribbon sample with rough edges was attached to the sample holder at one end and to the STM tip at the other end. During Joule heating and electron beam irradiation, carbon atoms at rough edges are vaporized to reconstruct smooth edges, which are either zigzag‐ or armchair‐edge configurations (Figure 4c,d). Joule heating and the preferred current flow along specific edges play a vital role for the reconstruction of edges. This method promises the efficient formation of all zigzag‐edge graphene nanoribbons.

Various graphene nanostructures with zigzag edges were fabricated from a top‐down approach. Patterned artificial defects (circular holes) were first created on graphene with e‐beam, then the defects serve as seeds for growth of different periodic nanostructures (honeycomb network, triangle islands, etc.), with zigzag edges (Figure 4e,f).^[^ ^46^ ^]^

Lithography (a top‐down approach) has been a widely used technique for producing graphene nanorobbons with well‐controlled edges. A mask is lithographically patterned on graphene, generally obtained by CVD. Then the parts that are not covered by the mask are removed by techniques such as plasma etching.^[^ ^47^ ^]^ The width of the nanoribbon obtained depends on the design of the mask, and the roughness of the edges depends on the resolution of the lithography and etching method. Nanoribbons can also be cut directly from graphene through scanning tunneling microscope lithography (STL), which is capable of patterning graphene through its tunneling current localized in a channel with lateral dimensions comparable to atomic width. Single‐layer graphene with either zigzag or armchair edges was prepared by STL on gold substrates (Figure 4g–j).^[^ ^48^ ^]^ STM is capable of imaging graphene at the atom resolution, which helps identify the zigzag or armchair directions. Fabrication of graphene nanoribbons with controlled width and edge structure was demonstrated in this study.

CVD method has also been used to produce graphene nanoribbons. A metallic catalytic template was used for the growth of nanoribbons. The size of nanoribbons is therefore dependent on that of the template.^[^ ^49^ ^]^ Detailed reviews of the different methods for producing graphene nanoribbons are available.^[^ ^50^ ^]^

## 5 Chemical Structure of GO and Its Correlation with Free Radicals

**Figure 5:**
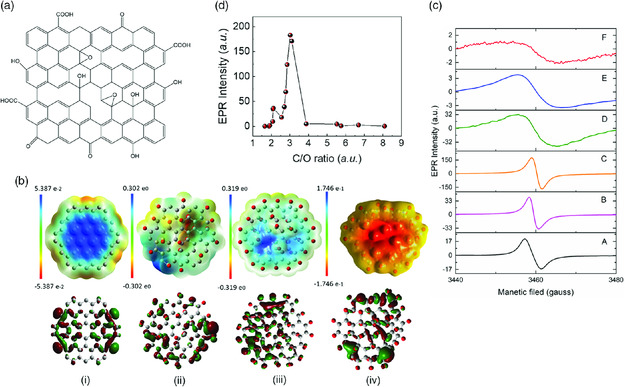
a) A schematic drawing of GO with radicals. b) Electrostatic potential mapping from charge density matrix for different samples, including i) pristine graphene, ii) partially oxidized GO (highest EPR), iii) highly oxidized GO, and iv) over oxidized GO. c) EPR spectra of samples obtained during progressive oxidation of graphite with KMnO_4_. d) Correlation of the EPR intensity of GO as a function of carbon to oxygen ratio. In part (c), from F to A, the oxidation degree is gradually increased; note that the *Y*‐axial scales of the spectra for each sample are different. The signals of poorly oxidized ones (F and E) are barely recognizable if drawn on the same scale. Reproduced with permission.^[53b]^ Copyright 2020, The Royal Society of Chemistry.

GO, produced by oxidative exfoliation of graphite, has inherent structural defects on its edges/holes and the basal plane, which are produced during oxidative cutting of graphene. It has multiple oxygenated groups, including carboxyl, hydroxyl, epoxide, and carbonyl groups (**Figure** **5**a).^[^ ^51^ ^]^ The contents of different oxygen groups depend on the preparation method and extent of oxidation, which affect the size and defects of GO.^[^ ^52^ ^]^ The oxygenated groups change the electron structure of graphene, for example, a carbonyl group adds an additional electron to its *π* system and an epoxy group removes two *π* electrons from the neighboring sublattice sites. The groups contribute to the formation of isolated *sp* ^2^ domains that change the electron distribution in the basal plane, and can lead to localization of unpaired electrons (radicals). Extensive oxidation can also create holes (internal edges) on the basal plane. Similar to the boundary edge, the geometry and chemistry of hole edges can contribute to the formation of edge state (radical‐like).^[21a]^ Reduction of GO increases defects which inherently increase the local electrochemical activity of graphene. However, a higher degree of reduction might not give a higher reactivity. Mildly reduced GO has demonstrated to have a higher content of radicals and reactivity.^[^ ^53^ ^]^ Its high electrocatalytic activity for energy conversion reaction has also been reported.^[^ ^54^ ^]^

The reactivity of GO has been explored for different applications such as chemical synthesis, antimicrobial uses, antioxidization, oxidizing degradation of pollutants, photo‐ and electrocatalysis for energy conversion reactions. Its reactivity has been generally attributed to its chemical structure, in particular the oxygenated groups. An increasing number of publications revealed that the electron structure, in particular the radicals on GO, contributes to its reactivity.^[^ ^54^, ^55^ ^]^ This indicates that it is feasible to produce reactive GO by engineering its radicals.

The existence of charge transfer between the oxygenated groups and *sp* ^2^ domains and reactive edge state on GO has been demonstrated in a limited number of studies.^[21a]^ To understand how the different oxygen groups and the degree of oxidation affect the electron structure (in particular, radicals), and to evaluate their relationship quantitatively are essential for developing efficient GO catalysts. Komeily‐Nia et al. used X‐ray photoelectron spectroscopy (XPS) and EPR to correlate the chemistry and radical property of GO during the progressive oxidation of graphite and the reduction of GO.[^7^, ^53^] In the process of progressive oxidation of graphite, the highest radical content was achieved (Figure 5c, sample C), followed by reduction in radicals with further oxidation (Figure 5c, samples B and A). For highly oxidized samples, the radical content can be increased by partial reduction. By combing the oxidation and reduction treatments, it revealed that a maximum radical content was achieved at a specific carbon to oxygen ratio (C/O) of about 3 (Figure 5d).^[53a]^ It was proposed that disturbance of the oxygenated groups to the chemical structure alters the electron distribution of the basal plane and edges, giving rise to localized unpaired electrons. DFT calculations demonstrated that pristine graphene has a very homogeneous charge distribution in its basal plane, while its edges have obvious uneven charge distribution (Figure 5b–i). This is in agreement with the high activity of the graphene edges.^[^ ^22^ ^]^ Partially oxidized graphene demonstrated uneven charge distribution even in the inner plane (Figure 5b‐ii, iii), while charge distribution on overoxidized graphene is quite homogeneous (Figure 5b‐iv). These results provide important information about the influence of oxygenated groups on the radicals, with good correlations, which indicates simple manipulation of C/O ratio can achieve highly efficient GO or rGO. However, how the defect structures (hole and edge geometries, vacancy) vary between the samples and how they contribute to the radical production also need to be understood. As known, reduction increases defects, which can contribute to the formation of more unpaired spins. The fact that when the reduction is above a certain degree, the radical content is reduced indicates that the oxygenated groups could play a role for stabilizing the radicals.

**Figure 6:**
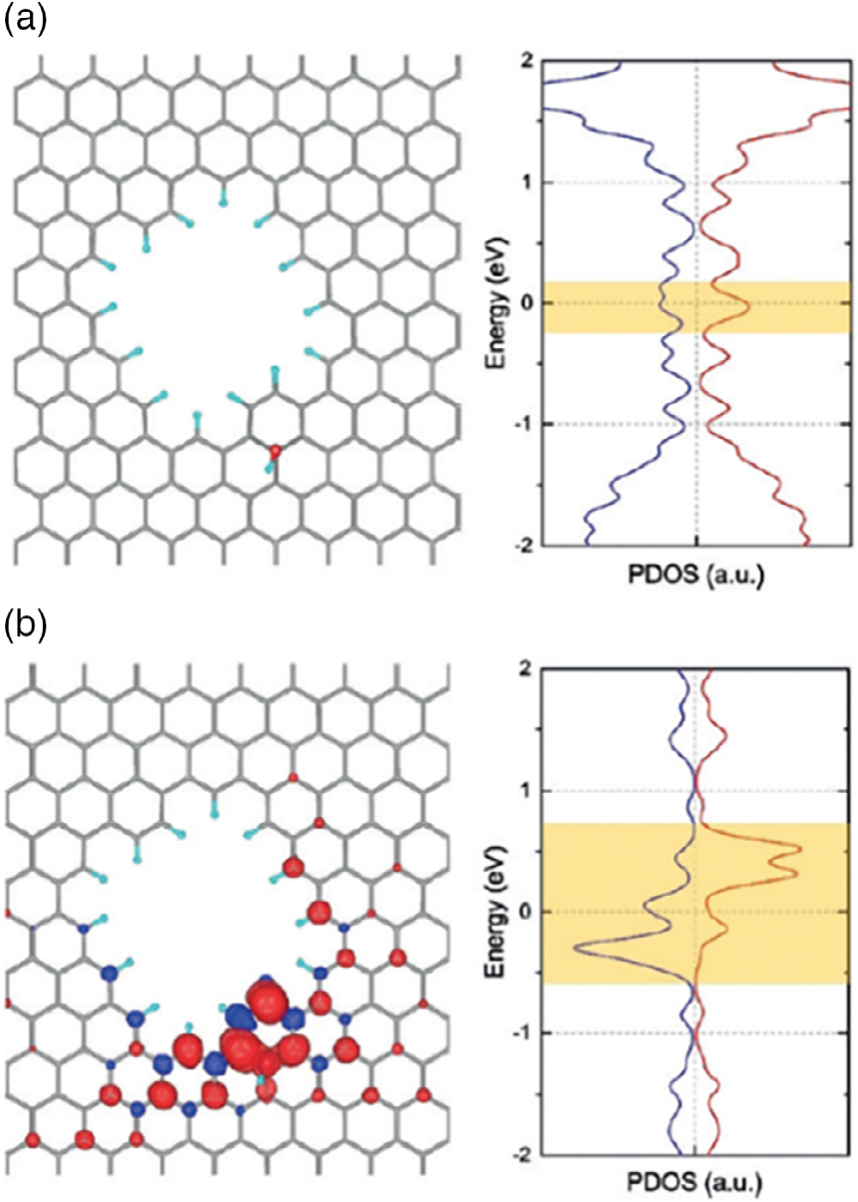
DFT calculations describing radical states with different models. Representation of the geometry (left) and projected density of states (PDOS) (right) of porous graphene: a) with —OH groups, and b) spin density (left) and PDOS (right) for 12 C atoms closest to the —OH group. Asymmetric spin‐up and spin‐down states are highlighted in yellow. Reproduced with permission.^[^ ^56^ ^]^ Copyright 2019, Wiley‐VCH GmbH.

A recent study that combines of experimental investigation and DFT calculations supports this hypothesis. It was found in the study that the hole defects and oxygenated groups are essential ingredients for the creation and stabilization of unpaired spins.^[^ ^56^ ^]^ When the —OH is located near the edges of the pores (**Figure** **6**a), the spin‐up and spin‐down densities turned out to be asymmetric, suggesting the source of the paramagnetism. The spins, generated in vacancies decorated with —OH functionalization, were localized near the functionalization site (Figure 6b). The presence of —OH on active sites near edges breaks the symmetry between the spin‐up and spin‐down electrons on the opposite edges, producing unpaired paramagnetic spins. A combination of experimental investigations with the localized probing techniques and more computer calculations is essential in future effort for achieving an in‐depth understanding.

## 6 Intrinsic Reactivity of GO and Its Applications: The Role of Free Radicals

Some studies have demonstrated that GO can be used alone (without chemical doping) for various applications, including initiators for polymerization, chemical bond formation/organic synthesis, antimicrobial and antioxidizing applications, as well as photo‐ and electrocatalysis, which are to be discussed here. Radical‐enhanced charge transfer has been proposed to be a mechanism.

### 6.1 Chemical Bond Formation

GO has been studied extensively for chemical bond formation since 2010. It is believed that the catalytic activity of GO originates from the oxygenated groups that make it an oxidant and acid catalyst for various reactions, including oxidation of sulfide, olefins, and different hydrocarbons, hydration,^[^ ^57^ ^]^ condensation,^[^ ^58^ ^]^ Friedel–Crafts addition,^[^ ^59^ ^]^ and aza–Michael additions.^[^ ^60^ ^]^ Detailed reviews on the GO‐based catalysts for carbon–carbon bond formation are available.^[^ ^61^ ^]^ In this section, only the studies that reveal the active role of radicals are covered. Compared with the oxygenated groups, the role of radicals in synthesis of small organic molecules has not been well studied. As discussed later, a few studies did indicate that radicals play a significant role in catalyzing bond formation.

**Figure 7:**
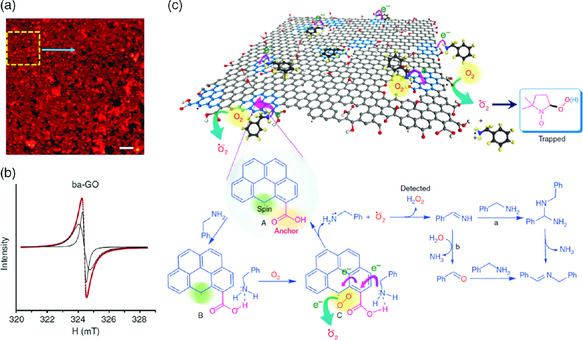
a) STM image of treated GO, b) EPR spectra, and c) proposed mechanism of the enhanced reactivity of ba‐GO catalyst. Reproduced with permission.^[^ ^62^ ^]^ Copyright 2012, Springer Nature.

It was observed that increasing its defects, the catalytic activity of GO for oxidative coupling of amines to imines was enhanced.^[^ ^62^ ^]^ A yield of 98% was achieved with a GO loading of 5 wt%. The catalyst was produced by sequential base and acid treatment of GO (ba‐GO), which creates a large number of nanovoids on GO (**Figure** **7**a). The edge of ba‐GO catalyst is rich in *π*‐electron radical, which exhibits fast spin–lattice relaxation through interaction with the adjacent *π*‐electron system (the broad linewidth in ESR of Figure 7b). It was believed that the active catalytic edge sites afforded enhanced kinetics for the trapping and activation of molecular oxygen by a sequence of electron transport and reduction to superoxide radicals (⋅O_2_^−)^ (Figure 7c), which stays surface‐bound to the GO to stabilize the positive charge of the holes. At the same time, the anchored amine is oxidized by positive charge of the hole to form the cation radical complex. The superoxide radical can abstract hydrogen atom from the amine radial cation to produce imine intermediates and H_2_O_2_. The carboxylic groups at the edges (including holes) of defects, along with the localized unpaired electrons, work synergistically to trap molecular oxygen and the amine molecules, which facilitates intermolecular arrangements. The catalyst exhibited excellent recyclability.

Morimoto et al. developed a reductive coupling of an aryldiazonium salt with a heteroaromatic compound using GO and rGO as catalysts and/or initiators.^[^ ^63^ ^]^ A yield as high as 68% could be achieved when the reaction took place at 40 °C for 6 h. The localized radicals on graphene played an important role in the catalysis. In addition, the material shows oxidative and reductive characters by controlling the amount of oxygenated groups and radicals.

The intrinsic catalytic activity of GO for direct CH—CH‐type coupling of xanthenes with arenes was investigated.^[^ ^64^ ^]^ Mechanistic studies involving molecular analogues, as well as trapped intermediates, were performed to probe the active sites. It was found that while the reaction was promoted synergistically by the C=O species and the zigzag edges in GO materials, the activity of the zigzag edges played a major role, due to their radical‐like structure. It was proposed that the ability of the materials to regenerate the zigzag edges, for example, by heating to remove some of the oxygenated groups, makes the catalyst reusable for multiple times.

### 6.2 Surface Polymerization of GO and Composites Formation

The radicals on GO have been used as initiators for either surface functionalization of GO or one‐step formation of polymer nanocomposites that involves in situ polymerization and cross‐linking with GO. Grafting polymers on the surface of GO modifies its dispersity in solvents and enhances its functionalities for applications.^[^ ^65^ ^]^

Using graphene‐based materials to enhance the mechanical properties, thermal stability/conductivity and electrical conductivity have attracted significant interest, as evidenced by the rapid increase in peer‐reviewed publications. Composites have been formed by different approaches, including 1) physical mixing,^[^ ^66^ ^]^ 2) in situ polymerization, which happens by mixing the 2D materials with monomers in the presence of radical initiators,^[^ ^67^ ^]^ 3) surface functionalization of graphene‐based materials, e.g., through the Diels–Alder (DA) reaction, to make them react with polymers,^[^ ^68^ ^]^ and 4) layer by layer assembly of polymers and graphene‐based materials.^[^ ^69^ ^]^ These will not be discussed in details as there have been many articles and review papers on the formation of composites.^[^ ^70^ ^]^ The various oxygenated groups of GO makes it advantageous over graphene for composites formation, as the groups facilitate functionalization and they can interact with polymers through hydrogen bonding. Therefore, GO has been more widely used for composites formation. GO dispersed in a polymer matrix can be reduced to get rGO/graphene to further improve the electrical conductivity of the composites. However, utilizing these oxygenated group does have some disadvantages as they can be removed when subject to heating, chemical exposure, light irradiation,^[7b]^ and so on. Polymers can also be grafted on the sp^2^ domain of carbon materials using radical polymerization, which needs radical initiators.^[^ ^71^ ^]^

**Figure 8:**
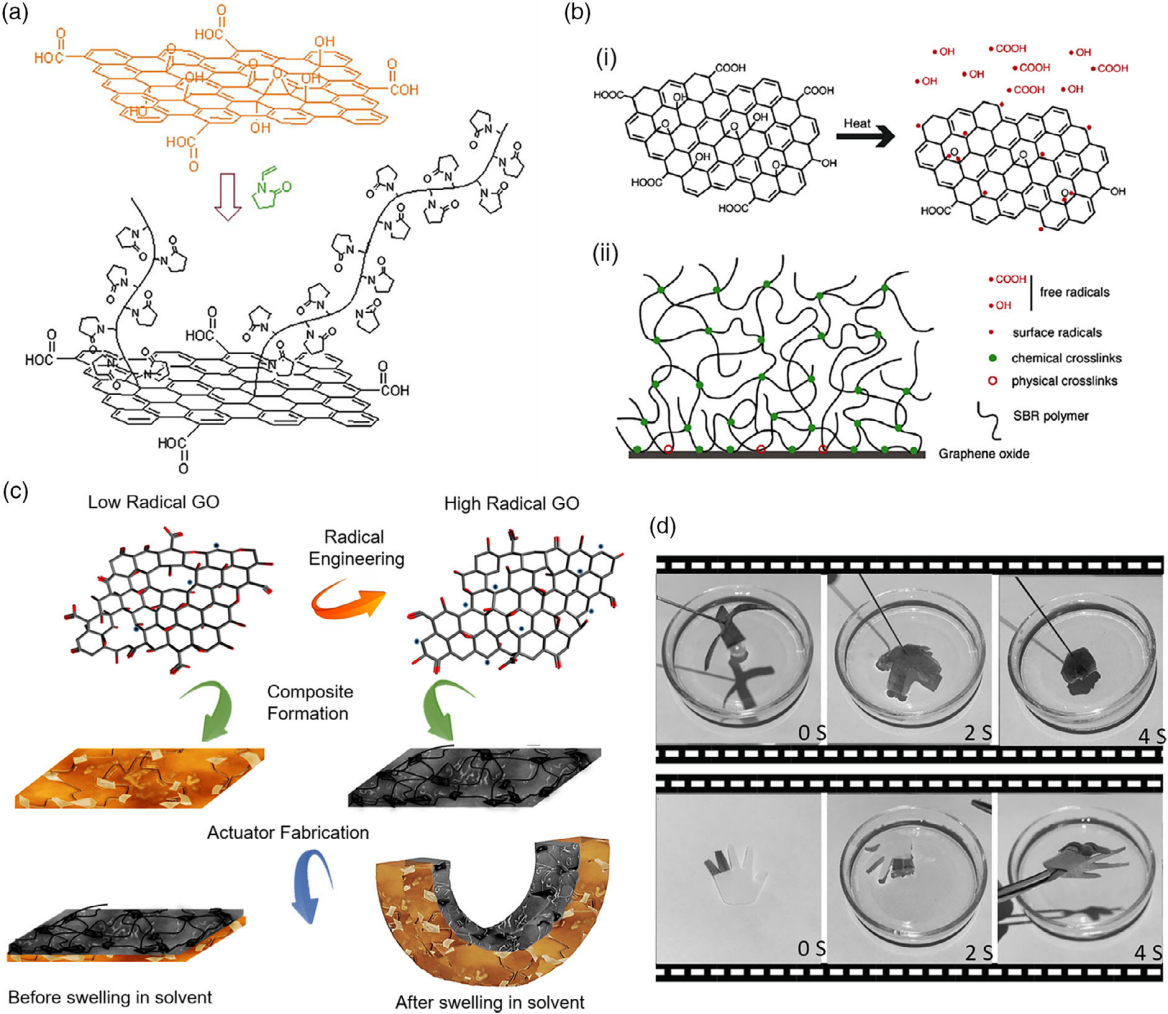
Free radicals of GO for surface polymerization and nanocomposites formation. a) Graft polymerization of N‐vinylpyrrolidone on GO initiated by the radicals at the defect sites of GO. b) A schematic description of the crosslinking mechanism of GO for SBR. c) Fabrication of bilayer polymer actuators using GO with different radical contents for the active and passive layers. d) Griping of an object in a solvent using a cross‐shaped bilayer actuator (the upper row) and folding fingers of an actuator. (a) Reproduced with permission.^[^ ^72^ ^]^ Copyright 2012, The Royal Society of Chemistry; (b) Reproduced with permission.^[^ ^74^ ^]^ Copyright 2017, Elsevier; (c,d) Reproduced with permission.^[55d]^ Copyright 2020, Elsevier.

The carbon radicals on GO offer a new approach for in situ polymer grafting and composites formation. The reactivity of the radicals eliminates the use of additional molecular initiators and makes stable functionalization. GO was used as an initiator for graft polymerization of N‐vinylpyrrolidone (NVP) (**Figure** **8**a). ^13^C‐NMR spectra indicated that breakage of weak bonds at the defects on the GO surface initialized the radical polymerization of NVP. The functionalized GO can be well dispersed in various solvents.^[^ ^72^ ^]^

Free radical grafting of styrene homo and copolymers on GO was reported.^[^ ^65^ ^]^ An intermediate, stearylamine‐modiﬁed graphite oxide (stearyl‐GO), containing both oleﬁnic unsaturation and stable graphene radicals, was used. “Grafting‐to” by addition of polystyrene radicals to graphene and “grafting‐from” by graphene‐initiated free radical polymerization afforded high graft yields. The in situ polymerization led to uniform graphene dispersion in the polymer matrix, enhanced elasticity, and markedly improved electrical conductivity and thermal stability of the nanocomposites.

Nanocomposites of poly(methyl methacrylate) (PMMA) and GO nanocomposites were formed by both thermal and GO radical‐initiated surface polymerization without an additional radical initiator. The grafting of PMMA chains to GO surfaces resulted in homogeneous dispersion of GO sheets in the PMMA matrix. At a GO loading level of just 0.5 wt%, the storage modulus of the nanocomposite was improved 14%, and the glass transition temperature was increased 12 °C in comparison with that of neat PMMA. Thermogravimetric analysis showed that the onset degradation temperature of the nanocomposite was increased 13 °C with a GO content of 0.25 wt%.^[^ ^73^ ^]^

Free radical and controlled radical polymerization of sodium 4‐vinylbenzenesulfonate with GO was reported.^[55b]^ The polymer obtained via reversible addition fragmentation chain transfer polymerization had a controlled molecular weight with a very narrow polydispersity (1.01–1.03). In another study, GO was used a filler and cross‐linker to produce mechanically robust styrene‐butadiene rubber (SBR).^[^ ^74^ ^]^ Upon heating, the homolytic cleavage of oxygenated groups on GO produces two classes of free radicals, which are free radicals (⋅OH, ⋅COOH) on cleaved groups and free radicals on GO (Figure 8b–i). The free radicals on the cleaved group diffuse away from GO, leading to cross‐linking of the rubber matrix; while the free radicals on GO are localized to induce interfacial crosslinking between GO and SBR (Figure 8b‐ii). The nanocomposite has better mechanical properties than SBR reinforced with conventional sulfur or dicumyl peroxide.

In the recent work of Komeily‐Nia et al., GO nanosheets with different radical contents were used to polymerize butyl acrylate.^[55d]^ It demonstrated that poly(butyl acrylate) can be grafted on the nanosheets, and the composites demonstrated different swelling behavior upon absorbing a solvent. A composite formed by GO with a higher radical content showed a less degree of swelling due to its denser cross‐linking. The nanosheets served as both radical initiators for polymerization and cross‐linkers for the composites formed. A bilayer actuator fabricated using two films with different degrees of swelling self‐folds when it absorbs a solvent (Figure 8c). Gripping of an object can also be achieved with actuators having a suitable structure (e.g., cross‐shaped) (Figure 8d). By slightly heating the bilayer structure, radicals promote polymerization of the residual monomers to cross‐link the two layers, creating strong interfacial binding between the two layers. This addresses the big debonding problem for layered polymer actuators.

It is noteworthy that apart from GO, fluorinated graphene (FG) has also been reported to be efficient initiators for radical polymerization in recent studies. FG has been an important precursor for the production of functionalized graphene (e.g., cyanographene and allyl‐graphene).^[^ ^75^ ^]^ Despite the strength of C—F bonds and high chemical stability of perﬂuorinated hydrocarbons, FG is very susceptible to reactions under ambient conditions, through nucleophilic substitution, which is accompanied by spontaneous defluorination.^[^ ^76^ ^]^ The exploration of its radicals and their applications is recent effort. During the fluorination process, which is initiated by the chemical defects on graphene, abundant spin/radical centers can form on FG.^[^ ^76^, ^77^ ^]^ The FG is able to induce radical polymerization and graft from polymerization of styrene monomers, leading to the formation of free polystyrene and polystyrene‐grafted graphene, and the initiation efficiency of FG was found to almost reach the level of commercial initiator azodiisobutyronitrile (AIBN).^[^ ^78^ ^]^ The radicals have also been used for grafting graphene with poly(acrylic acid) for lubrication applications.^[^ ^79^ ^]^ FG as a more recent catalytic material for radical polymerization is worth more detailed investigations. In particular, more work with the help of computer calculation is needed to reveal the mechanism of radical formation. Similar to oxygenation, fluorination also changes the charge distribution of graphene. However, fluorination does not bring about the severe damage (e.g., formation of holes) to the graphene plane as happens in the oxidation process. While it was observed that its spin density depends on the fluorine to carbon ratio, with the highest spin density achieved at a ratio of 0.4,^[^ ^77^ ^]^ how defluorination affects the radicals is also interesting to investigate, as FG is quite prone to defluorination in the presence of many common nucleophilic (dipolar) solvents, such as *N*,*N*‐dimethylformamide (DMF), dimethyl‐acetamide (DMAc), and *N*‐methyl‐2‐pyrrolidone (NMP), which are frequently used for chemical synthesis.^[^ ^80^ ^]^ In comparison, the radicals of GO is more sensitive to reducing agents. A comprehensive study on the radicals of the two materials is worthwhile.

Apart from reenforcing polymeric materials, graphene‐based materials can also slow down aging of the composites by their barrier (reduce oxygen permeability) and/or radical scavenging property.^[^ ^81^ ^]^ The ability of graphene‐based materials to scavenge various radicals such as hydroxyl and di(phenyl)‐(2,4,6‐trinitrophenyl) iminoazanium (DPPH) has been demonstrated.[^53^, ^82^] There have been many studies reported the radical scavenging and antioxidizing properties of GO, which are mainly attributed to the adduct formation at the electron‐rich *sp* ^2^ sites of GO and hydrogen donation from the oxygenated groups.^[^ ^82^, ^83^ ^]^ A detailed discussion will not be given in this review. Despite the extensive studies, correlating its efficiency with its radicals has been rarely reported. The study by Komeily‐Nia et al. indicate that the radical scavenging efficiency of GO is higher when it has more radicals.^[53a]^ Considering that the amount of radicals can be controlled conveniently through manipulating its C/O ratio, GO could be a class of novel antioxidant for important applications not limited to protection of polymers.

### 6.3 Photo‐ and Electrocatalysis for Energy Conversion Reactions

**Figure 9:**
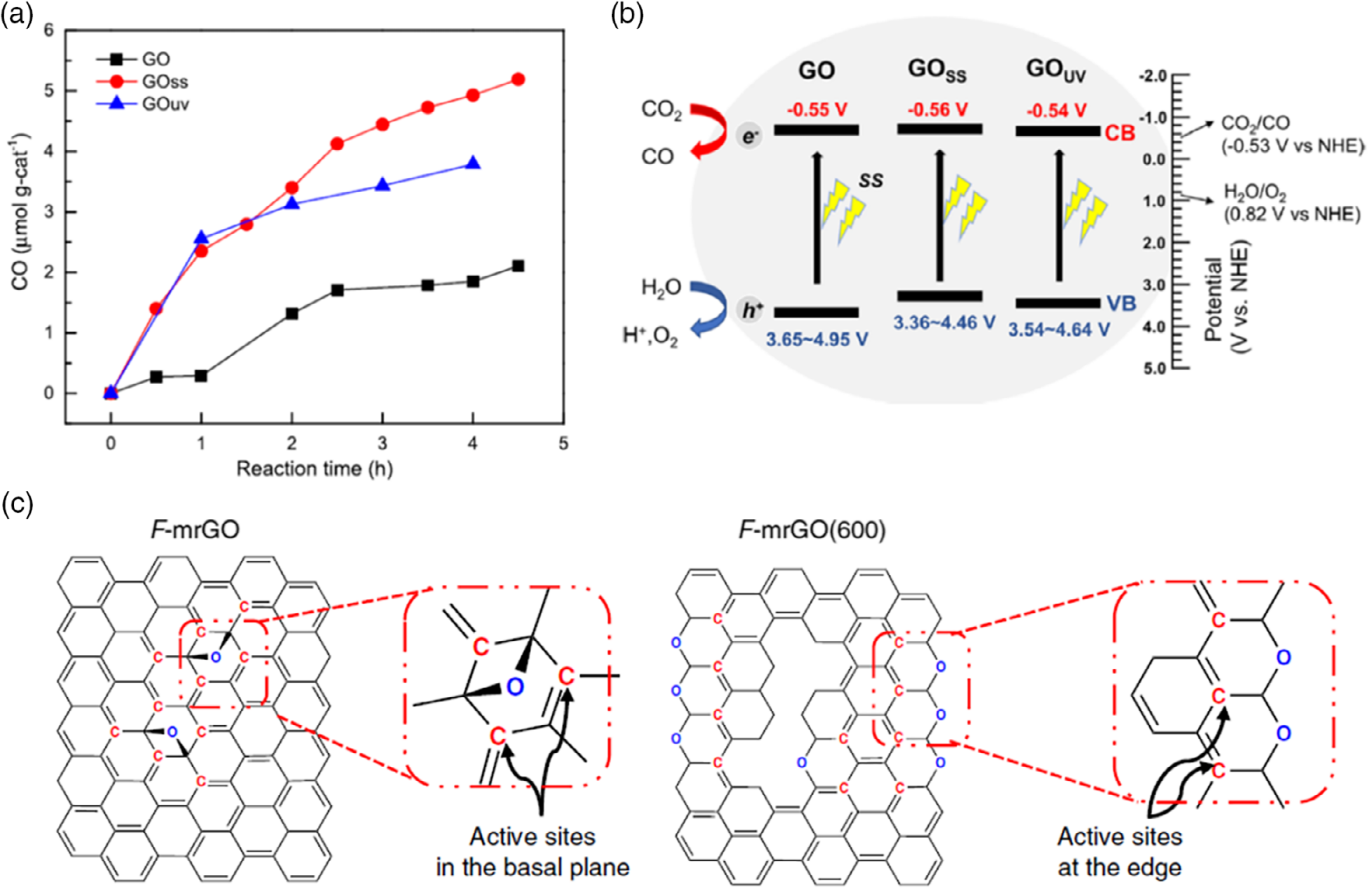
a) CO formation over time across GO, GOSS, and GOUV under simulated sunlight irradiation. b) Scheme of the photocatalytic CO_2_ reduction mechanism on GO and irradiated GO samples. c) Schemes of proposed low‐overpotential active sites on F‐mrGO and F‐mrGO(600). (a,b) Reproduced with permission.^[55a]^ Copyright 2020, The American Chemical Society; (c) Reproduced with permission.^[^ ^54^ ^]^ Copyright 2018, Springer Nature.

It was demonstrated in a very recent work that photoactivated GO could enhance the photocatalytic reduction of CO_2_.^[55a]^ GO was reduced with simulated light (GO_SS_) and UV light (GO_UV_), which were then used to reduce CO_2_. They demonstrated enhanced efficiency than pristine GO, with a CO yield (4 h reaction) ratio of 2.7:2.1:1 for GOss/GOuv/GO (**Figure** **9**a). ESR characterizations showed that the radical content of GO_SS_ (4.48 × 10^12^ spins (mg mL^−1^)^−1^) is higher than that of GO_UV_ (4.23 × 10^12^ spins (mg mL^−1^)^−1^), while GO (2.24 × 10^12^ spins (mg mL^−1^)^−1^) has the least radicals. This order of radical content corresponds to their defect densities. Irradiation during photocatalysis further increased the defects in the GO samples. The proposed mechanism is that light irradiation creates electron–hole pairs on the active sites, which are affected by *sp* ^2^ network and defects on the materials. The absorbed water molecules split to generate protons at the active sites of the holes, which function as acceptors because the valence bands of the materials are greater than the potential for theoretical water oxidation. Correspondingly, because the conducting bands of the materials are lower than the potential for CO_2_ reduction, the photogenerated electrons can act as donors to reduce absorbed CO_2_ to form CO (Figure 9b).

Mildly reduced GO (mrGO) has demonstrated highly selective and stable formation of peroxide at very low potentials (<10 mV) during electrocatalysis for ORR.^[^ ^54^ ^]^ Few‐layered mrGO (F‐mrGO) electrodes were fabricated and used for the catalysis. Further improvement of the performance of the mrGO was achieved by thermal annealing at 600 °C (F‐mrGO (600)). In situ Raman spectroelectrochemistry indicated that defects related to ether groups, such as epoxides, along the basal plane or at the sheet edges were active sites for HO_2_^-^ production. In a following theoretical study, it revealed that, apart from the edge and defects, the superior performance of mrGO was also attributable to the synergetic effects between the different oxygenated groups.^[^ ^84^ ^]^ As shown in Figure 5d, mildly reduced GO has a larger content of radicals, and the edges in particular zigzag edges also have more radicals than the basal plane. Though the radicals were not characterized in the studies, they should contribute to the catalytic activity of the materials. It is noteworthy that apart from the electronic properties of graphene‐based materials, their capabilities of absorbing substrates and reagents also contribute to their efficiency for catalytic applications.^[^ ^85^ ^]^

### 6.4 Catalytic Biosensing

The amphiphilic property of GO makes it a good material for biosensing. The oxygenated groups make GO dispersible in water. The hydrophobic aromatic structure and the hydrophilic groups contribute to the good affinity of GO for various biomolecules.^[^ ^86^ ^]^ The biosensors are generally based on ﬂuorescence resonance energy transfer (FRET), laser desorption/ionization mass spectrometry (LDI‐MS), surface‐enhanced Raman spectroscopy (SERS), and electrochemical detection. There have been many publications including review articles in this area.^[^ ^87^ ^]^ In comparison, sensing based on the catalytic activity of GO has been less reported. Among these studies, GO has been more generally used a cocatalyst or supporter to metallic or metal oxide catalysts.^[^ ^88^ ^]^

**Figure 10:**
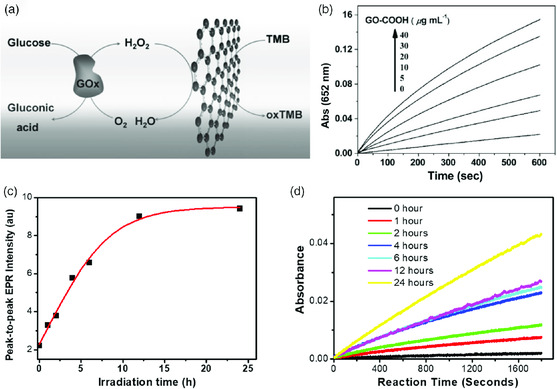
a) Schematic description of colorimetric detection of glucose by using GO. b) The time‐dependent absorbance changes at 652 nm in the absence (black) or presence of different concentrations of GO catalyst. c) Peak‐to‐peak intensity of the EPR spectra of GO as a function of ultraviolet B (UVB) irradiation duration. d) The time‐dependent absorbance change at 652 nm in the presence of nonirradiated GO and GO that was irradiated with UVB for different time. (a,b) Reproduced with permission.^[89a]^ Copyright 2010, Wiley‐VCH, GmbH; (c,d) Reproduced with permission.^[7a]^ Copyright 2013, The American Chemical Society.

The intrinsic activity of GO either in a free form or in a composite electrode for peroxidase‐like catalytic applications, which is useful for detection of biomolecules such as glucose and dopamine detection, has been reported.^[^ ^89^ ^]^ The activity of GO is attributed to the direct electron transfer to H_2_O_2_. The free form catalysis is generally based on colorimetric analysis. Kinetic studies showed that the efficiency of GO for catalyzing the reaction of peroxidase substrate 3,3,5,5‐tetramethylbenzidine (TMB) in the presence of H_2_O_2_ was even higher than the natural enzyme horseradish peroxidase (HRP) (**Figure** **10**a,b).^[89a]^ Intrinsic peroxidase‐like catalytic activity has also been observed for core–shell multiwalled CNT and GO nanoribbon.^[^ ^90^ ^]^ It was observed that when the GO nanoribbon was reduced, the efficiency of the core–shell structure was 5.9 times higher than that of MWCNTs and 8.4 times higher than that of the unreduced core–shell structure. The enhancement was attributed to the acceleration of the electron‐transfer process and the consequent facilitation of ⋅OH radical generation. The reduced structure also displays a higher affinity for both H_2_O_2_ and TMB than that of horseradish peroxidase, which contributes to faster electron transfer. Interestingly, the activity of GO to activate TMB was observed to correlate with its radical content.^[7a]^ As shown in Figure 10c, the EPR intensity of GO was increased by reduction with UV light irradiation, correspondingly, the absorbance of TMB was increased (Figure 10d). The advantages of nanomaterials‐based enzyme mimics include tunable catalytic activity, higher stability for long‐term storage, easiness of preparation, tolerance to harsh conditions, and low cost.

### 6.5 Catalytic Degradation of Environmental Pollutants

Graphene‐based materials have also been used for oxidative degradation of environmental pollutants such as phenols and pharmaceuticals (e.g., antibiotics). In this type of catalysis, sulfate radicals (SO_4_^• −^) are generally used for the oxidation of the pollutants, due to the high oxidative potential of these radicals, while the graphene‐based catalysts were found to promote the production of the sulfate radicals. Similar to other catalytic applications, element doping has been generally used to enhance the efficiency of the materials.^[^ ^91^ ^]^ rGO without any doping has demonstrated to be an efficient catalyst for activating peroxymonosulfate (PMS) to produce sulfate radicals.^[^ ^92^ ^]^ The efficiency is superior to that of transition metal oxide (Co_3_O_4_) in degradation of phenol, 2,4‐dichlorophenol, and a dye (methylene blue) in water. The proposed mechanism is donation of electrons from the zigzag edges of rGO. rGO has also been used as catalysts for oxidative desulfurization reactions to remove sulfur‐containing compounds from fuels.^[^ ^93^ ^]^ It was observed that the free radicals formed in the process played a significant role for the reactions. In the recent work of Komeily‐Nia et al., the efficiency of GO and rGO for degrading phenol was found to increase linearly with their radical contents.^[53b]^ The catalytic degradation of a pharmaceutical, acetaminophen, by PMS was also found to be promoted by biochar.^[^ ^94^ ^]^ Its catalytic activity increases with the graphitization degree and defects. The persistent free radicals of biochar were supposed to promote the activation of PMS. Interestingly, it was observed that the degradation of some pollutants could happen through either a radical or nonradical pathway, depending on the carbon nanostructure.^[^ ^95^ ^]^ For the nonradical pathway, PMS can oxidize them without creating sulfate radicals. A combination of experimental and DFT calculations indicated that the defective edges at the boundary of carbon network were able to facilitate the organic degradation without generation of the reactive radicals.^[^ ^95^ ^]^

### 6.6 Antimicrobial Applications

The antimicrobial property of graphene‐based materials has been demonstrated in many publications, e.g., it was reported that GO can treat multiple drug‐resistant bacterial superbugs isolated from infected patients, and its efficiency is much higher than some conventional antibiotics, offering hope for a last‐line antibiotic to address the global crisis of antimicrobial resistance.^[^ ^96^ ^]^ However, it has also been reported to have no antimicrobial properties in some studies.^[^ ^97^ ^]^ In fact, there are a big number of parameters that influence the antimicrobial testing results, which could explain the diverse results reported in the literature. The various influencing factors also contribute to the identification of multiple antimicrobial mechanisms.^[^ ^6^, ^98^ ^]^

The antimicrobial effects of graphene‐based materials depend on factors including the physical (size, shape/edge) and chemical properties of the materials,^[^ ^99^ ^]^ and the presence of impurities from the synthesis of the materials.^[^ ^97^ ^]^ The following antimicrobial mechanisms have been proposed: 1) physical insertion into cells, mainly attributed to the sharp edges of the materials,^[^ ^100^ ^]^ 2) extraction of lipids from cell membrane,^[^ ^101^ ^]^ 3) reactive oxygen species (ROS)‐dependent or independent (electron transfer) oxidation cellular contents,^[^ ^102^ ^]^ and 4) isolation of bacteria from its environment due to wrapping by graphene sheets.^[^ ^103^ ^]^ Depending on the properties of the materials, more than one bactericidal mechanism can be involved.^[102a]^ A few review articles are available for detailed information.^[^ ^6^, ^98^ ^]^ By functionalizing graphene or GO with antimicrobial peptides, enhanced membrane penetration and bactericidal efficiency have also been observed.^[^ ^104^ ^]^

Radicals can induce oxidation through the formation of ROS or by facilitating charge transfer, which contributes to bacteria killing. As discussed, unpaired/localized electrons are generally present on the defects of graphene materials. It is reasonable to assume that the graphene‐based materials that were used in different studies most probably have different levels of defects and thus radicals, due to the different methods/conditions for their preparation. GO produced for different studies can be different in C/O ratio, which correlates to its radical content. This could explain the different levels of toxicities reported in the literature.

**Figure 11:**
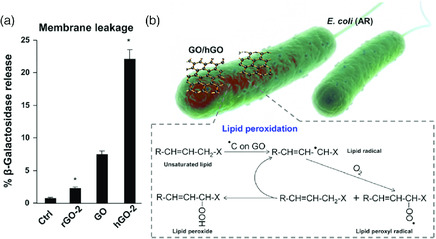
Influence of carbon radicals on GO on antimicrobial properties. a) β‐Galactosidase release from GO‐treated bacteria, b) illustration of the bactericidal effect of GO including membrane association and lipid peroxidation. hGO‐2 has the highest radical density, followed by GO and rGO‐2. Reproduced with permission.^[55c]^ Copyright 2016, The American Chemical Society.

Understanding how the radicals contribute to the toxicity of a graphene‐based materials is essential for illustrating the mechanisms related to antimicrobial efficiency or toxicity to cells, and it also helps the development of efficient antimicrobial agents. Encouragingly, it was observed that the bacterial killing ability of GO is primarily associated with the density of its carbon radicals.^[55c]^ The bactericidal action was supposed to occur through three steps: 1) electron transfer from the carbon radicals to the C atoms adjacent to double carbon bonds (C=C) in the lipid; 2) electron transfer to neighboring molecular dioxygens to create lipid peroxide radicals; and 3) formation of lipid peroxides (**Figure** **11**). Vertically aligned GO nanosheets with their edges in contact with bacterial were also observed to offer not only enhanced lipids disruption but also electron transfer to induce oxidation of glutathione in cells.^[102a]^

## 7 Conclusions and Outlook

Understanding how the physical (defects/edges) and chemical structure of graphene materials affect their electronic properties and intrinsic reactivity offers new opportunities for researchers to explore the huge potentials of these materials for various applications. The scope of application could be beyond those discussed in this review, for example, catalytic applications where graphene materials are used as cocatalysts could benefit from the enhanced electron transfer from the reactive graphene materials to the catalysts. The radical scavenging property also makes the graphene materials useful for protecting coatings from oxidation (antiaging) and the presence of radicals/charges shall be able to enhance materials with antistatic property.

The intrinsic reactivity of graphene materials relies on their electron structures, which depend on various parameters, including edge geometry, types and density of defects, and functionalization. The presence of radicals, an important aspect of the electron/chemical property of graphene materials, also depends on these parameters. As the intrinsic reactivity of these materials relies on electron transfer process, the role of unpaired electrons, which facilitate electron transfer, needs to receive more attention. Since the radical property can be more conveniently quantified with EPR technique, which gives an overall picture of the presence and content of unpaired electrons on a material, it offers a method for fast screening of reactive graphene materials. Defects and radicals are not two isolated properties of graphene materials. The creation of defects is frequently accompanied by the appearance of radicals. However, considering some defects may not give rise to radicals, both theoretical studies and experimental investigations are needed to elucidate how the different defects affect the radical property of these materials. The knowledge is essential for understanding the intrinsic reactivity of these materials and the mechanisms of enhancement by these materials. Developing methods that are capable of mass production of graphene materials with the defects crucial to the reactivity of the materials still needs significant efforts of researchers.

An increasing number of works have demonstrated that the intrinsic reactivity of graphene materials can be maximized by properly tuning their edges/defects, which can potentially eliminate the necessity for complicated element doping. Precise control over the edge geometry is currently limited to localized treatment (lithography). Looking for ways to mass produce graphene materials with a greater control over the edge geometry and defects shall be immediate research effort. In terms of GO, a number of studies have indicated that its reactivity positively and linearly correlates to its radical content. It is not clear if this also applies to other graphene materials (i.e., those not oxidized). The fact that edge‐rich (also rich in radicals) graphene has a higher reactivity supports this hypothesis. Despite the understanding achieved on the radical properties of GO, a clear understanding of the role of the distribution of the different oxygenated groups on GO in the formation of radicals and the stability/recyclability of the radicals for catalytic applications have not been achieved. In addition, how removal of the groups, as happens in the reduction process, changes the defect (holes, edges, and vacancy) structure of GO, also needs to be understood, which requires a combination of theoretical studies and experimental investigations particularly with the aid of localized probing techniques. The knowledge is not only essential for optimizing the reactivity and applications of GO, but also helps understand the mechanism of GO for promoting the reactions/applications. However, it worth mentioning that the radical content of GO is quite sensitive to its C/O ratio, which means that during its application, any factor during such as temperature (especially >100 °C), reducing agent and exposure to strong light shall affect its reactivity. Comprehensive studies on the reactivity and its stability from different origins such as edge, vacancy, and functionalization (oxygenation and fluorination, etc.) are needed for the development of reactive graphene materials suitable to different applications.

At the end, it is worth reemphasizing that understanding the intrinsic reactivity of graphene materials and developing capacity to control it will bring about significant advantages such as eliminating the use of cocatalysts, avoiding the necessity for element‐doping that is energy‐intensive and intricate to control, avoiding the use of toxic chemicals such as molecular initiators for polymerization, and reducing the use of molecular antibiotics which have caused the appearance of antibiotic‐resistant bacterial superbugs. These will facilitate the advance of carbon‐based, metal‐free catalysts and their applications in important energy and healthcare areas. These catalysts are not only low cost, less toxic to the environment, but also may offer a solution to the critical problems, e.g., the antibiotic resistance. With multiple antimicrobial mechanisms, it is unlikely for bacteria to develop resistance to this type of novel nanoantibiotic agent.

## Conflict of Interest

The authors declare no conflict of interest.
